# Deep learning applications in osteosarcoma MRI: A systematic review of recent advances in AI-based osteosarcoma diagnosis

**DOI:** 10.1371/journal.pone.0354896

**Published:** 2026-08-18

**Authors:** Galib Muhammad Shahriar Himel, Anusha Achuthan, Yusuf Abas Mohamed, Bee Ee Khoo, Mohd Ezane Bin Aziz

**Affiliations:** 1 School of Computer Sciences, Universiti Sains Malaysia, USM Penang, Malaysia; 2 School of Electrical and Electronic Engineering, Engineering Campus, USM Nibong Tebal, Penang, Malaysia; 3 Department of Radiology, School of Medical Sciences, Universiti Sains Malaysia, Kubang Kerian, Kelantan, Malaysia; Università degli Studi di Napoli Federico II: Universita degli Studi di Napoli Federico II, ITALY

## Abstract

Primary malignant bone tumours of the skeleton have a great diversity in their biological behaviour, and the most common in adolescence is osteosarcoma for which the diagnosis and therapeutic management are both a challenge. The use of machine and deep learning in image analysis for MRI, diffusion-weighted imaging (DWI) and dynamic contrast-enhanced MRI (DCE-MRI) to diagnose osteosarcoma has shown major improvements recently. This systematic review is performed according to PRISMA 2020 guidelines, and it comprises a critical analysis of 38 peer-reviewed articles from 2020 to 2025, which address the various aspects of tumour segmentation, classification, and prognosis. The methodological mapping revealed seven main categories that included numerous examples of radiomics (n = 8) and convolutional neural networks (CNNs, n = 6), as well as vision transformers and multimodal architectures for emerging technologies (n = 5 each). A quantitative evaluation of the segmentation performance showed that Multimodal achieved the best spatial overlap with an average Dice Similarity Coefficient (DSC) of 94.7% (range: 94.5–94.9%), while CNN of standard size was comparable, Lightweight networks had a DSC between 91.4 and 99.4% with an average of 91.9% and Vision Transformers a range of 90.5–94.9% with an average of 92.1%. By contrast, the performance gap among the Attention Mechanisms was greatest with a mean of 87.67% (64.6–98.5%), while the lowest was the mean 85.7% (81.6–89.8%) for Unsupervised Clustering. In addition to segmentation, the clinical nomograms based on radiomics also showed excellent prognostic potential:The AUC values for predicting pathological necrosis after chemotherapy varied from 0.807 to 0.848. More importantly it was found that there were significant translational gaps because all 38 models were limited to retrospective, single centre cohorts, with evaluation of risk of bias. Advanced algorithms perform well with localized precision but face a number of challenges with robust multicenter testing and a public repository.

## Introduction

Osteosarcoma is an aggressive primary bone cancer that mainly affects adolescents and young adults, accounting for roughly one fifth of all primary bone malignancies [1,2]. Because it often spreads early to the lungs and shows heterogeneous responses to treatment, it remains difficult to diagnose accurately and to manage optimally in everyday clinical practice. Careful evaluation of the true extent of the lesion, the response to neoadjuvant chemotherapy, and the risk of metastatic spread is essential for improving prognosis. Magnetic resonance imaging (MRI)—including advanced techniques such as diffusion weighted imaging (DWI) and dynamic contrast enhanced MRI (DCE MRI)—has become central for detailed tumor characterization, surgical planning, and post therapy assessment [3–5]. Nevertheless, visual assessment of these images is labor intensive and prone to differences between readers [6,7]. Over the past decade, computational methods based on machine learning (ML) and deep learning (DL) have advanced rapidly and shown strong performance in medical image analysis [8–11]. In osteosarcoma, such approaches have been applied to tasks including automated tumor segmentation [12], extraction of quantitative radiomic features [13], image based classification [14], and prediction of treatment response and patient outcome [15]. Convolutional neural networks (CNNs), transformer style architectures, hybrid pipelines, and more traditional algorithms such as support vector machines and random forests have all been explored to enhance and automate the interpretation of multi sequence imaging data. Although the number of publications in this field is growing quickly, the available studies remain scattered, and there is no unified overview of ML/DL techniques tailored specifically to osteosarcoma imaging. The purpose of this systematic review is to bridge this gap by providing a comprehensive summary of AI methods applied to MRI and related modalities in osteosarcoma. By systematically mapping current methodological trends, comparing model performance, and appraising clinical validity, this review aims to clarify the strengths and limitations of existing models. Ultimately, our objective is to identify persisting gaps and highlight future opportunities to move computer-assisted imaging of osteosarcoma closer to real-world clinical use. To address that the following Research Questions are formulated:

RQ1. Among studies published between January 2020 and December 2025, what machine learning and deep learning approaches have been applied to MRI and related modalities (e.g., DWI, DCE-MRI) for osteosarcoma diagnosis, tumor segmentation, and prognostic or treatment response prediction?

RQ2. How do different model families—such as conventional radiomics + ML, CNN based architectures, transformer based models, and hybrid frameworks—compare in terms of technical performance metrics (e.g., Dice, IoU, accuracy, AUC, sensitivity, specificity) across osteosarcoma MRI tasks?

RQ3. What are the key methodological characteristics and potential sources of bias in current AI-based osteosarcoma imaging studies (e.g., dataset size, single vs multi center design, internal vs external validation, retrospective vs prospective design, adherence to reporting standards of PRISMA?

RQ4. To what extent do existing AI models for osteosarcoma MRI incorporate clinically meaningful endpoints—such as histologic necrosis after neoadjuvant chemotherapy, RECIST measurements, overall survival, disease free survival, and metastasis—and what is their reported clinical utility?

RQ5. What cross cutting gaps, challenges, and future research directions emerge regarding data availability, multimodal imaging integration, computational efficiency, and real world clinical integration of AI systems for osteosarcoma imaging, especially in resource limited settings?

### Objectives

#### Primary objective.

OB_01. To systematically identify, synthesize, and critically appraise machine learning and deep learning approaches applied to MRI and related imaging modalities for osteosarcoma diagnosis, tumor segmentation, and treatment response/prognostic assessment, in studies published from January 2020 to December 2025.

#### Secondary objectives.

OB_02. To categorize included studies by imaging modality (e.g., conventional MRI, DWI, DCE MRI), model type (radiomics + ML, CNNs, transformers, hybrid models), and clinical task (segmentation, classification, response prediction, prognosis).

OB_03. To compare and summarize the reported technical performance of these models using standard metrics such as Dice coefficient, IoU, accuracy, AUC, sensitivity, specificity, precision, recall, and F1 score.

OB_04. To evaluate the methodological quality and potential risk of bias of the included studies, focusing on dataset characteristics, validation strategies (internal vs external), single vs multi center design, and transparency of reporting.

OB_05. To assess the extent of clinical translation, including how often AI models are linked to clinically relevant outcomes (histologic response to neoadjuvant chemotherapy, overall survival, disease free survival, metastasis) and whether they are tested or discussed within real world clinical workflows.

OB_06. To identify major limitations and future research priorities—such as data scarcity, lack of standardized public datasets, limited multimodal fusion, interpretability issues, and insufficient clinician involvement—and to provide recommendations for more clinically meaningful, scalable AI solutions in osteosarcoma imaging.

## Materials and Methods

This systematic review was designed to locate and synthesize research from the last six years that uses machine learning (ML) and deep learning (DL) methods on medical imaging—particularly MRI and related techniques—for the diagnosis, segmentation, and prognostic evaluation of osteosarcoma. The review followed the PRISMA (Preferred Reporting Items for Systematic Reviews and Meta-Analyses) guidelines, with clearly defined inclusion and exclusion criteria to structure the search and screening workflow. Journal articles were retrieved from five major bibliographic databases: Web of Science, Scopus, PubMed, IEEE Xplore and Embase, restricting the search to studies published between January 2020 and December 2025. The last search was done on 30th December, 2025.

**For Scopus, the search strategy was:**

TITLE(“osteosarcoma” AND “MRI”) AND (TITLE-ABS-KEY(“machine learning” OR “deep learning” OR “computer vision” OR “segmentation” OR “classification”)).

**For Web of Science, the corresponding query was:**

ALL=(Osteosarcoma AND MRI AND (Machine Learning OR Artificial Intelligence OR Computer Vision) AND (Segmentation OR Classification)).

**For PubMed, the query was:**

(“osteosarcoma”[ti] AND (“mri”[ti] OR “magnetic resonance imaging”[ti])) AND (“machine learning”[tiab] OR “deep learning”[tiab] OR “artificial intelligence”[tiab] OR “computer vision”[tiab] OR “segmentation”[tiab] OR “classification”[tiab] OR “radiomics”[tiab] OR “Osteosarcoma/diagnostic imaging”[Mesh] OR “Magnetic Resonance Imaging”[Mesh]).

**For IEEE Xplore, the corresponding query was:**

((“Document Title”:osteosarcoma AND (“Document Title”:MRI OR “Document Title”:"magnetic resonance imaging”)) AND (“Abstract”:*learning OR “Abstract”:"artificial intelligence” OR “Abstract”:"computer vision” OR “Abstract”:segmentation OR “Abstract”:classification OR “Document Terms”:*learning OR “Document Terms”:segmentation)).

**For Embase, the query was:**

(osteosarcoma:ti AND (mri:ti OR ‘magnetic resonance imaging’:ti)) AND (’machine learning’:ti,ab,kw OR ‘deep learning’:ti,ab,kw OR ‘artificial intelligence’:ti,ab,kw OR ‘computer vision’:ti,ab,kw OR segmentation:ti,ab,kw OR classification:ti,ab,kw OR ‘osteosarcoma’/exp OR ‘nuclear magnetic resonance imaging’/exp).

Although the syntax differs between databases, both queries were constructed to capture the same conceptual search space. Data handling and reference management were implemented in Python, which facilitated automated removal of duplicate records and systematic tracking of each step in the selection process. The initial database search identified 142 records. After de-duplication, 78 unique studies remained. So, the total number of papers selected for screening was 78. Titles, abstracts, and methods sections were then screened to exclude papers that did not focus on osteosarcoma, did not use MRI or closely related imaging modalities, or did not apply ML/DL-based approaches. Following this screening phase, 38 studies were retained for full-text assessment and after assessing them all 38 papers were included in the review study.

### Selection Process

All records retrieved from the database searches were exported and screened in two stages. In the first stage, one reviewer independently screened titles and abstracts to remove clearly irrelevant citations based on the predefined inclusion and exclusion criteria. Disagreements were resolved through discussion, with a 2nd reviewer consulted when necessary. In the second stage, the same reviewers independently assessed the full text of potentially eligible articles to confirm eligibility. Python scripts were used only to assist with technical tasks such as deduplication and tracking study identifiers and were not involved in decision making about study inclusion.

Studies were excluded if they met any of the following criteria: published before 2020, reviews or editorials, or not employing machine or deep learning methods directly relevant to imaging-based osteosarcoma analysis. Inclusion criteria required the use of ML or DL techniques applied to osteosarcoma diagnosis, segmentation, classification, or response prediction using MRI, DWI, or other clinical imaging modalities. The inclusion and exclusion criteria are described in Table 1.

**Table 1:** Inclusion and exclusion criteria for studies.

Category	Inclusion criteria	Exclusion criteria
**Population / disease focus**	Studies where osteosarcoma is the primary disease of interest, or where imaging datasets are explicitly labeled as osteosarcoma.	Records not focused on osteosarcoma, or mixed tumor cohorts where osteosarcoma specific results cannot be separated.
**Imaging modality**	Studies using MRI or closely related MRI-based modalities, including conventional MRI, DWI, and DCE MRI, for osteosarcoma imaging.	Studies not based on MRI or related imaging modalities (e.g., purely CT/X ray/PET without MRI, or non imaging work).
**AI / analytical approach**	Use of machine learning (ML) or deep learning (DL) methods applied to osteosarcoma imaging for diagnosis, segmentation, classification, radiomics, or treatment/response prediction.	Studies not utilizing ML/DL, or using ML/DL methods not directly relevant to imaging based osteosarcoma analysis.
**Publication characteristics**	Peer reviewed journal or conference original research articles; published between January 2020 and September 2025; full text available.	Articles published before 2020, or non–peer reviewed material.
**Study type / document type**	Empirical studies reporting experimental or clinical results for ML/DL models on osteosarcoma imaging.	Reviews, editorials, letters, commentaries, or purely theoretical papers without implemented ML/DL experiments.

A backward and forward citation search method, as proposed by Webster and Watson (2002), was also conducted to ensure comprehensiveness [16]. After completing all stages of screening and selection, a total of 38 studies were included in the final review. Data related to models, imaging modalities, datasets, evaluation metrics, and clinical outcomes were extracted for qualitative synthesis and comparison.

### Data Collection Process

Data extraction was performed using a piloted extraction form. For each included study, one reviewer extracted all relevant information and a second reviewer independently checked the entries against the original article. Any inconsistencies were resolved by consensus. Where important information (e.g., dataset characteristics, details of ground truth annotation, or numerical performance metrics) was missing or unclear, we relied on information provided in the main text, figures, and supplementary material; study authors were not routinely contacted for additional data.

### Data Items Outcomes

For each included study, we extracted outcome data related to model performance. For segmentation tasks, primary outcomes were the Dice Similarity Coefficient (DSC) and Intersection over Union (IoU), with secondary metrics including Hausdorff distance, sensitivity, and specificity where reported. For classification, detection, and prognostic modelling tasks, we extracted accuracy, area under the receiver operating characteristic curve (AUC), F1 score, sensitivity, specificity, and other task specific metrics. When multiple models or experimental settings were presented in a single article, we prioritised the authors’ main or best performing configuration, while reporting alternative results narratively when they provided additional insight.

### Data Items and Other Variables

In addition to outcomes, we extracted descriptive variables for each study, including sample size, patient population (e.g., age range, tumor site), imaging modality and sequence (e.g., T1 weighted, T2 weighted, DWI, DCE MRI), study design (retrospective vs. prospective; single centre vs. multi centre), dataset source and splitting strategy (training/validation/test), AI methodology (e.g., radiomics, CNNs, transformers, hybrid frameworks), ground truth annotation procedures, and any reported external validation. Information on the original study’s funding and declared conflicts of interest was recorded when available. When critical details were not explicitly reported (for example, whether splitting was slice wise or patient wise), we coded them as “not reported” rather than inferring from context.

### Effect Measures

In this review, model performance metrics were treated as the primary effect measures. For segmentation studies, DSC and IoU were considered the main effect measures, complemented by Hausdorff distance, sensitivity, and specificity where available. For classification and prognostic models, the principal effect measures were accuracy, AUC, and F1 score, with additional reporting of sensitivity and specificity when provided. Effect measures were extracted and presented as reported in the original articles without transformation.

### Data Synthesis Methods

Owing to substantial heterogeneity in imaging protocols, AI model architectures, dataset characteristics, and reported metrics across the included studies, we did not plan or conduct a quantitative meta analysis. Instead, we used a narrative synthesis approach. Studies were initially grouped according to their primary aim (e.g., tumor segmentation, radiomics based prognostic modelling, treatment response prediction, decision support systems) and then stratified by methodological class (traditional machine learning, convolutional neural networks, transformer based models, and hybrid frameworks). Within each group, we tabulated key study characteristics and main performance metrics in summary tables (e.g., Tables 1–3) and compared results descriptively using ranges and, where meaningful, median values. We did not impute missing data. Potential sources of heterogeneity, such as sample size, imaging sequence, presence of clinical variables, and type of validation (internal vs. external), were explored qualitatively in the narrative synthesis, with specific patterns highlighted in the “Current Trends and Applications” and “Limitations and Future Directions” sections. No formal sensitivity analyses, subgroup analyses, or meta regressions were performed because no pooled quantitative synthesis was conducted.

**Table 2:** Dataset description.

Ref No.	Dataset Name / Source	Availability	Dataset Type	Patients	Images / Slices
[18]	Post-DAF Preprocessed MRI	Private	MRI	204	>80,000 images (63,783 valid)
[19]	Multi-modality Osteosarcoma Dataset	Private	MRI (T1WI, T2WI, SUB)	40	3,255 slices
[20,28,40]	Monash University Research Dataset	Private	MRI	204	>4,000 images
[21,32,34,53]	Xiangya Hospital Dataset	Private	MRI	204	>80,000 images
[22]	Data from Second Xiangya Hospital, China	Private	Medical Imaging (MRI) and Clinical Data	136	10–40 slices per sequence
[23]	T1 MRI (Fat Segmentation)	Private	T1-weighted MRI	43	Not specified
[24]	Data from AIIMS, India	Private	Medical Imaging (MRI) and Clinical Data	40	Not specified
[25]	Public Osteosarcoma MRI dataset	Public	Retrospective medical imaging data	Not specified	50 scan images
[26]	HUSM Osteosarcoma Dataset	Private	MRI (T1, T2, T1_FSE+GADO)	7	135 slices
[27]	Osteosarcoma DWI MRI Dataset	Private	DWI MRI	55	3,520 images
[29,35,38]	Three Hospitals Dataset (China)	Private	MRI	204	>80,000 images
[30]	Clinical MRI from Xiangya Hospital	Private	MRI	204	81,326 images
[31]	MRI + Index Data (Second Xiangya Hospital)	Private	MRI with clinical indexes	198	>70,000 images
[36]	Clinical Osteosarcoma MRI	Private	MRI with manual annotations	Not specified	>4,000 images
[37]	Huaihua Hospitals Dataset	Private	MRI	210	>4,000 images
[39]	Clinical MRI from Xiangya + Mobile Lab	Private	MRI	>200	>80,000 images
[42]	Osteosarcoma MRI (multiple hospitals)	Private	Multiparametric MRI + Clinical Data	78	Not specified
[43]	First Affiliated Hospital of Sun Yat-Sen University	Private	Medical Imaging (MRI) and Pathological Data	12	127 tissue-level ROIs
[44]	Osteosarcoma dataset	Private	Retrospective imaging and clinical data	102	Not specified
[45]	Multicenter osteosarcoma dataset	Private	Retrospective imaging and clinical data	102	Not specified
[46]	Istanbul University dataset	Private	Retrospective clinical dataset	21	Not specified
[47]	Dr. Soetomo Hospital Dataset	Private	MRI (DWI, DCE-MRI)	43	Not specified
[48]	Toronto Oncology Center Dataset	Private	T2-weighted MRI	105	Not specified
[49]	MRI for NAC Prediction	Private	DWI + Clinical	209	Not specified
[50]	DCE-MRI Multicenter Dataset	Private	DCE-MRI	85	Not specified
[51]	Osteosarcoma MRI (Monash University)	Private	MRI	Not specified	>4,000 images
[52]	Osteosarcoma MRI (Second Xiangya Hospital)	Private	MRI	286	>80,000 images
[54]	Partner Hospitals Dataset	Private	MRI	40	274 images
[55]	Monash University Dataset	Private	MRI	204	5,000 images

**Notes:** MRI = Magnetic Resonance Imaging; DWI = Diffusion-Weighted Imaging; DCE = Dynamic Contrast-Enhanced; NAC = Neoadjuvant Chemotherapy; ROI = Region of Interest.

**Table 3:** Risk of bias analysis.

No.	Study	Design / Sample	Participants RoB	Predictors RoB	Outcome RoB	Analysis RoB	Overall RoB
MS01	[18] Zhong 2024	Retrospective; large multi-hospital MRI dataset; SR + segmentation + decision support	Moderate	Moderate	Moderate	High	High
MS02	[19] Wu 2024	Retrospective; moderate single-centre multi-modality MRI (~40 pts)	Moderate	Moderate	Moderate	High	High
MS03	[20] He 2023	Retrospective; mixed multi-cancer MRI; very small osteosarcoma subset	High	Moderate	Moderate	High	High
MS04	[21] Liu 2022	Retrospective; large osteosarcoma MRI dataset from tertiary centres	Moderate	Moderate	Moderate	High	High
MS05	[22] Zhou 2024	Retrospective; 136 osteosarcoma pts, multiparametric MRI + clinical follow-up	Moderate	Moderate	Moderate	High	High
MS06	[23] Shukor 2023	Retrospective; small single-centre cohort (~43 pts)	High	Moderate	Moderate	High	High
MS07	[24] Kayal 2020	Retrospective; small single-centre DWI cohort (~40 pts)	High	Moderate	Moderate	High	High
MS08	[25] Nasor 2021	Retrospective; 50 MRI scans, public dataset; no clinical data	High	Moderate	Moderate	High	High
MS09	[26] Othman 2022	Retrospective; extremely small HUSM dataset (~7 pts, 135 slices)	High	Moderate	Moderate	High	High
MS10	[27] Baidya Kayal 2020	Retrospective; 55 pts, pre-treatment DWI (single centre)	High	Moderate	Moderate	High	High
MS11	[28] Wei 2023	Retrospective; large Monash MRI dataset (>4,000 images, ~200 pts)	Moderate	Moderate	Moderate	High	High
MS12	[29] J. Wu 2022	Retrospective; large osteosarcoma MRI dataset; assistant segmentation system	Moderate	Moderate	Moderate	High	High
MS13	[30] Wang 2022	Retrospective; large osteosarcoma MRI dataset from Chinese centres	Moderate	Moderate	Moderate	High	High
MS14	[31] J. Wu 2022	Retrospective; > 70,000 MRI images, ~198 pts; multi-stage pipeline	Moderate	Moderate	Moderate	High	High
MS15	[32] Wu 2022	Retrospective; large osteosarcoma MRI dataset (>80,000 images)	Moderate	Moderate	Moderate	High	High
MS16	[33] Jia 2022	Retrospective; multi-cancer dataset; only one osteosarcoma case	High	Moderate	Moderate	High	High
MS17	[34] Wu 2022	Retrospective; clinical osteosarcoma MRI (>4,000 images)	Moderate	Moderate	Moderate	High	High
MS18	[35] Wu 2022	Retrospective; > 80,000 post-processed MRI images (~204 pts)	Moderate	Moderate	Moderate	High	High
MS19	[36] Gou 2022	Retrospective; > 4,000 MRI images (~210 pts) from Huaihua hospitals	Moderate	Moderate	Moderate	High	High
MS20	[37] Tang 2022	Retrospective; > 70,000 MRI images with clinical indices (~198 pts)	Moderate	Moderate	Moderate	High	High
MS21	[38] Shen 2022	Retrospective; ~80,000 clinical MRI samples (~204 pts)	Moderate	Moderate	Moderate	High	High
MS22	[39] Lv 2022	Retrospective; large osteosarcoma MRI dataset from tertiary centre(s)	Moderate	Moderate	Moderate	High	High
MS23	[40] Lv 2023	Retrospective; three-hospital MRI dataset (>80,000 images, ~204 pts)	Moderate	Moderate	Moderate	High	High
MS24	[41] Zhou 2022	Arbitrary comparison; no single patient-level dataset	NA	NA	NA	NA	NA
MS25	[42] Luo 2022	Retrospective; 78 pts, multi-parametric MRI from multiple hospitals	Moderate	Moderate	Low	Moderate	Moderate
MS26	[43] Huang 2020	Prospective pilot; 12 pts, 127 tissue-level ROIs	High	Moderate	Low	High	High
MS27	[44] Zhang 2021	Retrospective; 102 pts from 4 hospitals, DCE-MRI	Moderate	Moderate	Low	Moderate	Moderate
MS28	[45] Chen 2021	Retrospective; 102 pts from 4 hospitals, CE-FS T1WI	Moderate	Moderate	Low	Moderate	Moderate
MS29	[46] Yildirim 2022	Retrospective; 21 pts, single centre	High	Moderate	Low	High	High
MS30	[47] Setiawati 2023	Retrospective; 43 pts, single centre	High	Moderate	Low	High	High
MS31	[48] White 2023	Retrospective; 105 pts, single tertiary centre	Moderate	Moderate	Low	Moderate	Moderate
MS32	[49] Zhang 2024	Retrospective; 209 pts from 2 centres	Low	Moderate	Low	Moderate	Moderate
MS33	[50] Kalisvaart 2024	Retrospective; 85 pts, 2 centres (train + external test)	Moderate	Moderate	Low	Moderate	Moderate
MS34	[51] Liu 2022	Retrospective; > 4,000 annotated MR images (~200 pts)	Moderate	Moderate	Moderate	High	High
MS35	[52] Ouyang 2022	Retrospective; > 80,000 MRI images (~204–286 pts)	Moderate	Moderate	Moderate	High	High
MS36	[53] Ling 2022	Retrospective; > 80,000 MRI images with expert labels (~204 pts)	Moderate	Moderate	Moderate	High	High
MS37	[54] Zou 2023	Retrospective; relatively small MRI set (~40 pts, ~200–300 images)	High	Moderate	Moderate	High	High
MS38	[55] He 2023	Retrospective; ~5,000 MRI images (~204 pts); “small-sample” focus	Moderate	Moderate	Moderate	High	High

**Notes:** RoB = Risk of Bias; SR = Super-Resolution; pts = patients; NA = Not Applicable.

**Fig 1:**
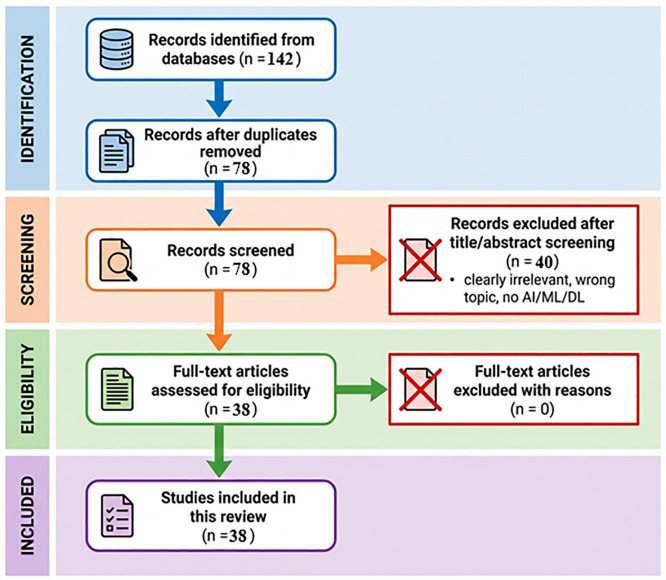
PRISMA framework.

The full search and filtering strategy is visually summarized in Fig 1 using the PRISMA flow diagram [17] format.

Given the diversity of study designs, target tasks, and reported outcome measures, we did not conduct a quantitative meta-analysis or compute pooled effect estimates. Instead, a narrative synthesis was undertaken. Studies were grouped according to their primary AI application (tumour segmentation, lesion classification, radiomics-based prediction, treatment-response or prognostic modelling, and multi-modal decision-support systems). Within each group, we qualitatively compared key effect measures such as Dice coefficient, IoU, AUC and accuracy, but no formal measures of statistical heterogeneity or sensitivity analyses were performed.

Because of the substantial heterogeneity in imaging protocols, model architectures, outcome definitions and reporting standards across the included studies, a single formal risk-of-bias tool (such as QUADAS-2 or PROBAST) could not be uniformly applied. Instead, we qualitatively appraised study quality by considering factors such as dataset size, clarity of inclusion and exclusion criteria, handling of train–validation–test splits, use of external or cross-institutional validation, and completeness of reporting. A formal GRADE assessment of certainty of evidence was not undertaken; consequently, the interpretations provided in this review should be regarded as a qualitative synthesis of the current evidence base. One reviewer worked on this process.

For each study that met the inclusion criteria, two authors independently extracted data using a pre-piloted spreadsheet. Extracted variables included: first author, year of publication, country or region, sample size, imaging modality and MRI sequence type (e.g., T1-weighted, T2-weighted, DWI, DCE-MRI), primary AI task (segmentation, classification, detection, radiomics, or prognosis), model architecture (e.g., CNN, U-Net, transformer or hybrid networks), dataset characteristics (public vs. private, single- vs. multi-centre), and reported performance metrics (e.g., Dice coefficient, IoU, accuracy, AUC, sensitivity, specificity), as well as any available clinical outcomes such as treatment response or survival. Extracted data were cross-checked and any inconsistencies were resolved by consensus.

Study selection and screening were performed in line with PRISMA 2020 recommendations. After automatic duplicate removal, one reviewers independently screened titles and abstracts to identify potentially eligible records. Full texts of all candidate articles were then assessed in duplicate. Any disagreements at either stage were resolved through discussion and, when necessary, consultation with a third senior reviewer. Reasons for exclusion at the full-text stage (for example, non-osteosarcoma population, non-MRI imaging, lack of ML/DL methodology, or abstract-only reports) were documented to ensure transparency.

A detailed PRISMA flow diagram of the study selection process is presented in Fig 1.

## Various Approaches

**Fig 2:**
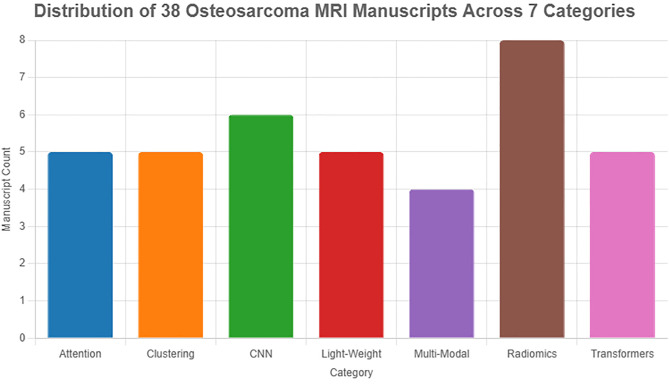
Various approaches taken in 38 papers.

The 38 studies used in this paper were distributed in total 7 categories. Those categories are demonstrated in Fig 2.

### Attention Mechanism

**Fig 3:**
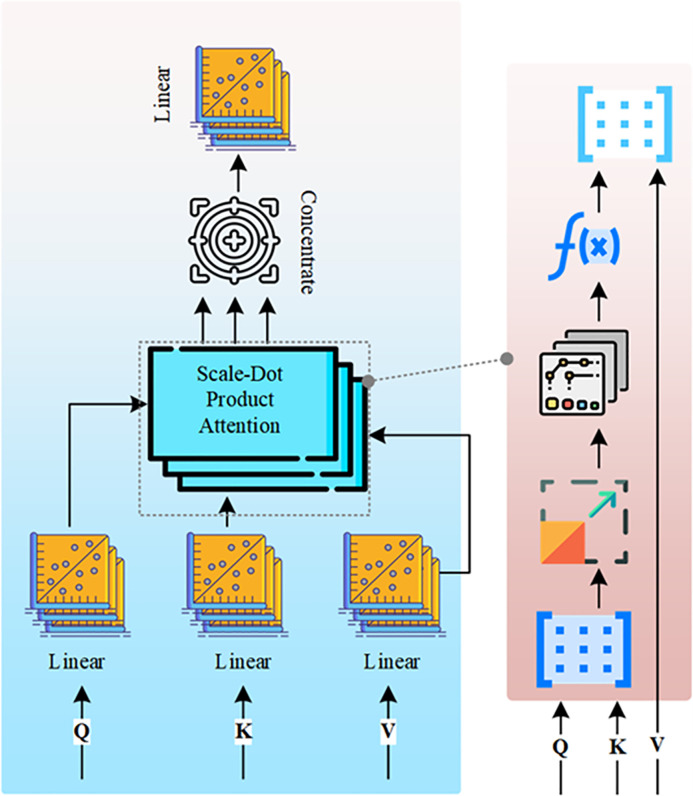
Attention mechanism.

Attention is a mechanism in machine learning, particularly in neural networks, that allows the model to focus on specific parts of the input data while processing it. Inspired by human cognitive attention, it dynamically assigns different weights to different elements to prioritize the most relevant information. Attention mechanisms have become fundamental in transformer architectures, significantly improving performance in tasks like natural language processing (NLP) and computer vision. Fig 3 represents the basic attention mechanism diagram.

Zhong et al. [18] describe an advanced MRI based framework for osteosarcoma assessment that combines image super resolution with deep neural networks to improve both image clarity and tumor segmentation. Their approach is built on a fully convolutional network enhanced with attention components, allowing the model to focus on both broad contextual structure and fine local detail, which helps to more precisely trace tumor borders. To reduce overfitting, they apply extensive data augmentation and regularization, and evaluate performance with cross validation across multiple clinical datasets. They also incorporate transformer style modules to capture longer range contextual relationships within the images, which further strengthens diagnostic accuracy and suggests good potential for real world clinical use. Wu et al. [19] introduce DECIDE, a decoupled network that treats semantic region labeling and boundary detection as related but distinct tasks for osteosarcoma MRI segmentation. The model integrates information from multiple MRI sequences and combines side outputs from different layers to generate the final tumor contour. By feeding multi scale inputs into the encoder, the method preserves details across different spatial resolutions, while an embedded attention mechanism fine tunes feature extraction. Together, these design choices improve segmentation performance, produce more reliable tumor edge definition, and offer stronger support for clinical decision making in osteosarcoma management. He et al. [20] developed a new segmentation framework for osteosarcoma MRI that combines a Y shaped network structure with EfficientNet encoders. A specially designed regularization term is applied to the attention masks so that the model concentrates on tumor tissue while still sampling the surrounding context. By exploiting multi scale feature maps and finely tuned attention modules, their method sharpens tumor edges and boosts both accuracy and stability in automatic lesion delineation. Liu et al. [21] introduced an attention preserving segmentation scheme for osteosarcoma MRI, in which convolutional neural networks are coupled with tailored attention blocks to strengthen multi scale feature capture and to better outline small or subtle tumor foci. The pipeline includes automatic pre processing, structured feature extraction, and dual supervised outputs, which together reduce the impact of artifacts and limited receptive fields. This setup lightens the imaging workload for radiologists and supports faster, more reliable clinical decision making for patients with osteosarcoma. Zhou et al. [22] designed a three stage workflow that links advanced convolutional segmentation, radiomic analysis, and clinical data integration for osteosarcoma assessment. Using multiparametric MRI, they first perform self supervised segmentation to obtain accurate tumor masks. High dimensional imaging descriptors are then extracted and combined with clinical variables, while iterative optimization and attention modules are used to refine the model. Tests on multiple datasets show that this strategy outperforms conventional approaches, providing more precise segmentation and stronger prognostic insight to guide osteosarcoma management.

The provided material synthesizes research on osteosarcoma MRI segmentation, illustrating significant methodological advancements from attention-based mechanisms for small target detection to complex multi-modality feature fusion and integration with clinical prognosis. These studies collectively report consistent performance gains over baseline U-Net architectures, with key improvements such as the CaPaN model’s 2% higher precision for small targets, the MPFNet’s 84.19% mean Dice Similarity Coefficient (DSC) for multi-parameter MRI, and the DECIDE network’s balanced achievement of 70.40% Dice and 74.85% precision. Despite these advancements, the studies demonstrate a shared limitation regarding the lack of comparative analysis across different approaches, often constrained by limited external validation and an incomplete discussion of computational efficiency trade-offs.

### Clustering-based Approach

**Fig 4:**
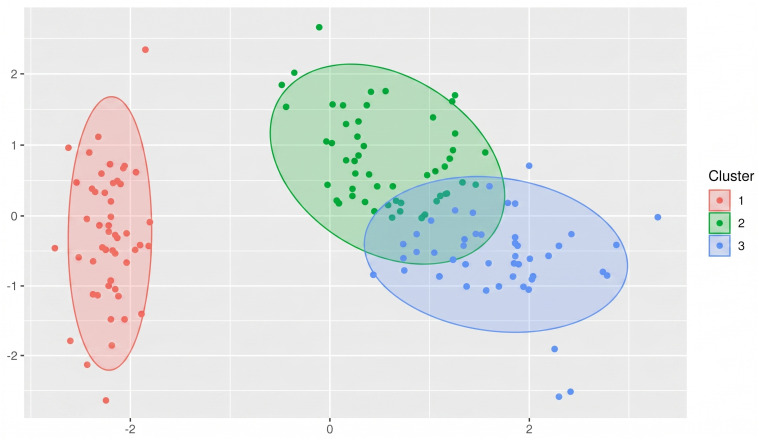
Clustering approach.

Clustering is an unsupervised machine learning technique used to group similar data points together based on their features. Unlike classification, clustering does not rely on predefined labels—instead, it identifies inherent patterns or structures in the data. Common clustering algorithms include K-means, hierarchical clustering, and DBSCAN. Applications range from customer segmentation in marketing to anomaly detection in cybersecurity. Fig 4 represents an example of simple clustering.

Muhammad Shukor et al. [23] presented a segmentation method for osteosarcoma on T1 weighted fat suppressed MRI using active contour models. In their scheme, an adaptively initialized mask is allowed to evolve under an energy minimization process so that the contour gradually aligns with the interface between fat, muscle, and tumor. Before and during this evolution, Gaussian filtering and entropy based thresholding are used to suppress noise and sharpen edges, improving boundary localization. When tested against expert manual segmentations, the method showed strong agreement while markedly cutting down the time and effort required from radiologists, leading to more consistent assessments of osteosarcoma images. Kayal et al. [24] developed an automatic segmentation pipeline based on diffusion weighted MRI to outline osteosarcoma lesions. Their approach combines Simple Linear Iterative Clustering superpixels (SLIC S) with Fuzzy C Means clustering, producing Dice similarity coefficients of roughly 70–83%. By avoiding manual placement of regions of interest, the method helps to mitigate low signal to noise issues that commonly affect diffusion imaging. Quantitative analysis of apparent diffusion coefficient (ADC) values was then used to verify segmentation quality and to compute RECIST style tumor measurements, with statistical testing demonstrating significant associations between imaging derived metrics and clinical indicators. Nasor et al. [25] proposed a fully automatic MRI segmentation strategy for osteosarcoma that chains together several classical image processing steps. The process starts with K means clustering to separate intensity groups, followed by Chan–Vese level set segmentation to refine the tumor outline. Additional Gaussian smoothing and Canny edge detection are applied to further clean and stabilize the contours, and morphological operations with object counting are used to remove spurious regions and merge relevant tumor areas. Evaluated on a set of 50 MRI exams, the workflow produced stable results across different sequences and heterogeneous textures, supporting its use as a reliable tool for clinical assessment. Haizan Othman et al. [26] introduced an automated segmentation method tailored to three thigh MRI sequences—T1, T2, and contrast enhanced T1_FSE+GADO—for osteosarcoma evaluation. Their algorithm combines Otsu’s global thresholding with Fuzzy C Means clustering to separate tumor tissue from surrounding structures. The segmented areas and perimeters were compared with reference annotations, showing good agreement. By addressing problems such as overlapping gray level distributions and reducing reliance on manual outlining, the method improves reproducibility and accuracy of tumor measurements and is well suited for fast integration into routine reporting. In a separate study, Kayal et al. [27] systematically compared nine different segmentation techniques on diffusion weighted MRI for osteosarcoma delineation. The comparison included unsupervised methods such as Otsu thresholding and region growing, alongside more advanced approaches like active contour models. Their framework focuses on extracting quantitative image descriptors—covering texture, signal intensity, and shape—to obtain robust definitions of tumor borders despite low signal to noise ratio and marked lesion heterogeneity. This comparative work provides a structured basis for selecting segmentation strategies for diagnostic work up, prognostic modeling, and treatment response monitoring in oncologic imaging.

This category details the evolution of osteosarcoma segmentation from early algorithm comparisons to sophisticated hybrid and sequence-specific automated systems. Methodological improvements, such as the hybridization of K-means, Chan-Vese, and morphological operations, have yielded accuracy gains of 16–19% over single-algorithm approaches, achieving accuracies as high as 98.02%. Advantages of these automated systems include increased clinical efficiency—with RECIST scoring and segmentation tasks reduced to mere seconds—and the mitigation of subjective observer bias inherent in manual interpretation. However, significant gaps persist, notably the technical difficulty of mask initialization in Active Contour models and challenges with low signal-to-noise ratios or intensity inhomogeneities. Furthermore, these studies are limited by small cohort sizes, a lack of prospective clinical trial validation, and significant methodological inconsistencies across MRI sequences, leaving a critical translation gap between technical performance and actual integration into radiologist workflows.

### Convolutional Neural Networks

**Fig 5:**
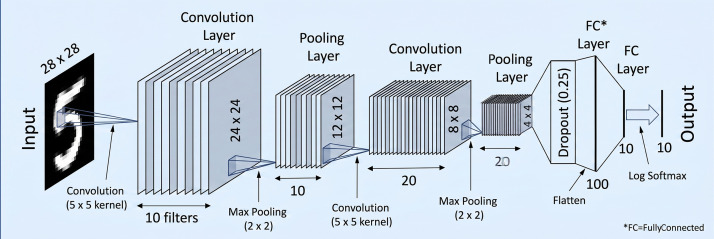
CNN architecture.

A Convolutional Neural Network (CNN) is a specialized deep learning architecture designed for processing structured grid-like data, such as images. CNNs use convolutional layers to automatically detect spatial hierarchies of features, from edges and textures to complex objects. Key components include convolutional filters, pooling layers, and fully connected layers. CNNs are widely used in image recognition, medical imaging, and autonomous driving due to their efficiency in capturing local patterns. Fig 5 represents the base CNN diagram.

Wei et al. [28] present a new MRI based tumor segmentation framework that relies on class correlation pattern aggregation to support clinical decision making. Their network uses convolutional feature extractors and fusion modules arranged so that samples from the same category are pulled together in the representation space. By combining rich feature encodings with tailored aggregation operations, the method sharpens tumor boundary depiction. When compared with established architectures such as U Net, FCN, and FPN, the framework achieves more accurate and stable segmentation of osteosarcoma while keeping computational cost under control. Wu et al. [29] propose DecoupleSegNet, an auxiliary segmentation model designed for osteosarcoma MRI. The network separately learns a coarse structural representation and a fine grained edge representation, producing two complementary feature maps. These are merged through a 1×1 convolution to strengthen contour information that is critical for precise tumor outlining. This decoupled design improves sensitivity to subtle intensity differences between tumor and surrounding tissue, reducing the need for manual corrections and delivering higher segmentation quality in practical use. Wang et al. [30] describe a supporting segmentation pipeline for osteosarcoma MRI that couples noise suppression with local image enhancement. The process begins with a powerful denoising stage to remove acquisition artifacts, followed by locally adaptive contrast enhancement to highlight faint lesion margins. The preprocessed images are then subjected to automated segmentation to extract accurate regions of interest for further quantitative assessment. Comparative experiments show that this workflow is faster and more consistent than conventional manual contouring, helping to improve treatment evaluation. Wu et al. [31] design a full MRI analysis scheme for osteosarcoma that links three main components: image pre screening, noise reduction, and lesion segmentation. First, an automatic screening step discards slices without diagnostic value. Next, a refined non local means algorithm, accelerated using integral images, is employed to suppress noise. Finally, a convolutional network performs tumor segmentation on the cleaned images. This end to end pipeline reduces computational burden, improves image quality, and provides reliable tumor masks, thereby increasing the usefulness of MRI examinations in routine practice. In another study, Wu et al. [32] introduce BA GCA Net, a segmentation network that embeds boundary aware grid contextual attention to more accurately localize osteosarcoma on MRI. The method is designed to capture both fine local textures and broader contextual cues by adaptively varying patch sizes. A padding–crop strategy is used to alleviate information loss between adjacent patches, which is especially important in areas with indistinct tumor margins. Dedicated boundary preserving components further protect edge information, lowering manual editing requirements and enhancing consistency in busy clinical environments. Jia et al. [33] develop AIMIS3D, a hardware independent platform that combines multimodal image registration, segmentation, and 3D visualization. Their system uses a deep convolutional network based on YOLOv3 to segment organs and tumor structures with high spatial precision. Before segmentation, imaging data are standardized through preprocessing and normalization. The resulting 3D volumes can then be rendered at high resolution for printing or interactive review, supporting preoperative planning and patient education. The interface allows clinicians to manipulate the 3D models—rotating, translating, zooming, and adding annotations—via gestures or voice commands, which facilitates clearer explanation of disease status to patients and families.

This synthesis of recent research highlights the shift toward advanced, highly accurate, and computationally efficient deep learning models for osteosarcoma MRI segmentation, with newer frameworks achieving up to 96% accuracy. Key methodological innovations across these studies include class-correlation pattern aggregation, body-edge feature relationship modeling, boundary-aware attention mechanisms, and transformer integration, all of which demonstrate scalability and clinical potential through large-scale validations using datasets ranging from 4,000 to over 80,000 images. Despite these technical achievements, the field suffers from a significant standardization deficit, as there are no shared benchmarks or protocols to facilitate meaningful performance comparisons. Furthermore, high dataset heterogeneity across institutions and the absence of prospective clinical trials limit the generalizability and practical clinical deployment of these methods, while the narrow focus on osteosarcoma restricts broader medical application.

### Light Weight

A lightweight machine learning model is designed to run with very small demands on computation, memory, and runtime, so that it can be used directly on devices with modest hardware, such as phones or embedded systems. Methods such as pruning, quantization, and knowledge distillation are commonly applied to shrink the model while keeping its predictive performance almost unchanged. Such compact models are particularly important in real time scenarios where processing must be fast and hardware resources are limited.

Wu et al. [34] developed a residual fusion network specifically aimed at segmenting osteosarcoma on MRI scans when computing power is restricted. The network uses residual links together with multi scale feature fusion, and adds supervised side outputs so that both high level semantic information and fine structural details are learned. This architecture substantially cuts down the number of parameters and computational cost but still maintains high accuracy. Tests on clinical MRI datasets showed stable, reliable segmentation results, and repeated cross validation indicated good generalization, suggesting that the method is well suited for hospitals in developing regions. In a related study, Wu et al. [35] designed an intelligent segmentation framework built around a feature extraction module and a hierarchical feature fusion strategy based on feature pyramid networks (FPN). The system simultaneously captures low resolution features rich in semantics and high resolution features containing detailed spatial information from different levels of the network. Each fused level is then used to make its own prediction, which differs from conventional single head designs. This two stage structure lowers computational burden while preserving accurate delineation of osteosarcoma lesions on MRI, and evaluations on clinical data showed consistently strong and reliable performance. Gou et al. [36] proposed AIMSost, an attention driven MRI segmentation system for osteosarcoma. Instead of standard convolutional blocks, they introduced a custom module that combines residual connections, LayerNorm, and depth wise convolutions, which decreases computation while maintaining segmentation quality. Drawing inspiration from AttendSeg, AIMSost boosts tumor contour accuracy, raising Dice and IOU metrics by more than 2.79% and 2.03%, respectively. This improvement supports earlier lesion identification and more informed clinical decision making, with the method remaining efficient and robust across different imaging conditions. Tang et al. [37] presented a real time MRI segmentation pipeline for osteosarcoma that couples sophisticated denoising with precise tumor localization. The workflow first applies a pre Eformer model to suppress image noise, then uses nonparametric localization and enhancement techniques to highlight suspicious regions. An adaptive segmentation network subsequently merges local contextual cues with global information to outline tumor borders accurately. This strategy improves segmentation performance and is intended to support automated diagnostic workflows and treatment planning. Shen et al. [38] introduced an MRI based osteosarcoma segmentation system built on a guided aggregated bilateral network. Their approach combines edge detection with bilateral filtering to sharpen tumor margins and reduce noise. Using a highly compact model, the system was able to process more than 80,000 clinical MRI images with high segmentation precision. It reliably distinguishes tumor tissue from surrounding anatomy, enabling fast image analysis and stable diagnostic support in routine clinical use.

These five studies contribute a robust suite of deep learning frameworks—including SepUNet with conditional random fields, multiscale residual fusion networks, attention-based systems, and guided bilateral networks—specifically tailored to address osteosarcoma MRI segmentation challenges in resource-constrained environments. A significant methodological strength across these works is the successful balance achieved between high segmentation accuracy (consistently reaching 0.95) and lightweight, computationally efficient architectures suitable for primary care settings. By effectively addressing multi-scale feature extraction challenges and reducing manual diagnosis time and observer bias, these models offer a promising advancement for clinical decision support. However, a major gap remains in the field’s standardization; the lack of shared evaluation protocols, identical baseline comparisons, and common metrics prevents a definitive ranking of these approaches. Furthermore, the generalizability of these findings is constrained by the reliance on datasets exclusively sourced from Chinese hospitals, highlighting an urgent need for external validation across diverse global populations and medical institutions.

### Multimodal Approach

A multimodal dataset brings together information from several different channels or data types—for example text, images, audio, or sensor readings—so that models can learn from their combined strengths. In a medical context, this might mean linking MRI scans with written clinical notes and laboratory measurements, allowing algorithms to form a more complete picture of a patient and perform better on demanding tasks such as diagnosis or treatment planning.

Lv et al. [39] describe a multiscale tumor localization strategy for osteosarcoma MRI that merges a range of segmentation backbones—including MSFCN, MSRN, FCN, FPN, and U-Net—to capture lesions over different spatial resolutions. Their framework uses prior information to guide the network, incorporating surrounding anatomy and contextual cues to refine the tumor mask. By limiting manual input and improving generalization, the method yields stable, accurate segmentations that can support earlier detection and more precise treatment planning in routine practice. In a related study, Lv et al. [40] present an image‐analysis system for clinical decision support that combines structurally designed multiscale residual blocks with a symmetric U shaped segmentation architecture. By using convolutional kernels of various sizes, the network is able to emphasize fine boundary details while also preserving broad contextual structure, which leads to more reliable delineation of pathological tissue and more efficient workflows in radiology departments. Zhou et al. [41] report a convolutional neural network–based pipeline for automated evaluation of bone tumors that covers preprocessing, segmentation, and classification, and is designed to handle multiple imaging modalities such as CT, MRI, and radiography. Deep convolutional filters are used to capture both local textural patterns and global morphology, while transfer learning and augmentation strategies are applied to strengthen performance on limited datasets. This framework improves diagnostic consistency and aids clinical decision making, but the authors also emphasize the current shortage of large, well curated public datasets dedicated specifically to bone tumors, which remains a key obstacle for data driven approaches. Building on multiparametric MRI, Luo et al. [42] constructed a predictive model for synchronous lung metastases in osteosarcoma by combining radiological features from T1 weighted, T2 weighted, and contrast enhanced T1 weighted scans with clinical variables. Radiomic descriptors of tumor texture, intensity, and morphology were extracted and then filtered using LASSO regression to retain only the most informative features, enabling development of a robust tool for early identification of metastatic risk and for supporting individualized prognosis assessment.

These four studies provide convergent evidence for the effectiveness of AI across the osteosarcoma diagnostic workflow, from initial detection to the prediction of synchronous lung metastases. Methodological innovations include prior-guided few-shot learning and edge-enhancement with transformer-based networks, both of which achieve high segmentation performance, with Dice coefficients reaching 0.945 and 0.949 respectively. Furthermore, radiomics analysis has demonstrated superior clinical utility, with AUC values for metastasis prediction reaching 0.957. Collectively, these models offer distinct advantages, such as eliminating subjective manual interpretation, saving labor and time, and enhancing predictive accuracy—for instance, Lv et al. [40] achieved 4.3% higher performance than existing models while reducing resource consumption. However, the field continues to face shared limitations: these models remain highly dependent on image quality and specific acquisition protocols, are often constrained by limited dataset sizes for training, and require significant computational resources that can hinder accessibility in resource-limited settings. Most critically, a significant gap remains between high-performing research models and their actual implementation in clinical practice, underscoring the need for larger validation studies to bridge this translation deficit.

### Radiomics

Osteosarcoma focused MRI radiomics refers to the extraction of large numbers of quantitative descriptors from MRI scans of bone tumors, with the goal of supporting diagnosis, prognostic assessment, and treatment planning. By coupling medical imaging with machine learning methods, radiomics can uncover imaging patterns that are not obvious on visual inspection, helping to characterize tumor biology, anticipate treatment response, and move toward more individualized management.

Huang et al. [43] carried out a pilot study combining diffusion weighted, T2 weighted, and contrast enhanced subtraction MRI to estimate tumour necrosis after neoadjuvant chemotherapy in limb osteosarcoma. Using random forest classifiers trained on standardized ADC values and signal intensities from co registered tissue samples, they showed that multi parametric MRI clearly outperformed ADC alone in distinguishing viable from non viable tumour components, suggesting a practical route towards non invasive, preoperative necrosis assessment. Building on dynamic contrast imaging, Zhang et al. [44] developed a radiomics nomogram based on three dimensional DCE MRI of osteosarcoma to evaluate the effectiveness of neoadjuvant chemotherapy. From over a thousand texture, shape, and enhancement related features, a compact radiomic signature was derived and combined with clinical variables, yielding a prediction model that more accurately separated good from poor responders than conventional imaging or clinical factors alone. In a multicentre cohort, Chen et al. [45] constructed an MRI based radiomics signature using contrast enhanced fat suppressed T1 weighted images acquired before treatment. A large set of intensity, texture, and wavelet features was reduced using penalised regression, and the selected descriptors were fed into a classifier to predict pathological response to neoadjuvant chemotherapy. The resulting model achieved high discriminatory performance across hospitals, indicating that pretreatment MRI contains sufficient quantitative information to anticipate histologic outcome. Yildirim et al. [46] focused on more conventional MRI metrics in a retrospective series of patients treated at a single institution, comparing tumour size, intramedullary extension, and the thickness of the most avidly enhancing component before and after chemotherapy. While changes in overall volume and marrow extent did not correlate reliably with histologic response, a significant reduction in the largest enhancing solid component was associated with better Huvos grades, supporting this parameter as a practical imaging surrogate of treatment effect. Setiawati et al. [47] investigated how diffusion and perfusion characteristics vary among osteosarcoma histopathologic subtypes using ADC maps and time–intensity curve analysis from DCE MRI. By placing multiple regions of interest within each tumour, they demonstrated subtype specific patterns: chondroblastic lesions showed the highest mean ADC values, whereas osteoblastic and small cell tumours exhibited steeper enhancement slopes and greater maximum enhancement. Significant correlations between ADC, dynamic enhancement metrics, and histology suggest that combined diffusion–perfusion profiling may help refine subtype diagnosis and monitor disease evolution. White et al. [48] used a radiomics strategy based on T2 weighted fat suppressed sequences to estimate histologic response to chemotherapy, overall survival, and disease free survival in patients with high grade intramedullary osteosarcoma. Quantitative features were derived from fast spin echo STIR images to capture intratumoral heterogeneity, after careful preprocessing, segmentation, and analysis conducted in line with contemporary radiomics standards and TRIPOD reporting guidance. The study received ethical approval, and redundant variables were reduced through correlation based filtering, allowing construction of stable predictive models that show promise for guiding individualized therapy. In a separate retrospective cohort of 209 patients from two institutions, Zhang et al. [49] evaluated response to neoadjuvant chemotherapy by stratifying patients according to the percentage of tumor necrosis at histopathology. Both clinical variables and imaging metrics, including apparent diffusion coefficient (ADC) values from diffusion weighted MRI, were extracted after meticulous region of interest definition. Feature selection combined variance thresholding, SelectKBest, and LASSO regression, and the resulting feature set was incorporated into machine learning models that were ultimately presented as a nomogram, improving the accuracy and clinical utility of response prediction. Kalisvaart et al. [50] proposed a dynamic contrast enhanced MRI–based approach to predict neoadjuvant chemotherapy response in osteosarcoma. Tumor tissue was segmented using both whole slab and focused area schemes, enabling derivation of kinetic parameters such as wash in rate, time to initial enhancement, and area under the enhancement curve. Texture features were further analyzed, and LASSO regression was applied to identify informative predictors, while ROC analysis with Youden’s index was used to define optimal cut off values. To address inter center variability, ComBat harmonization was employed during external validation. Nonetheless, modest sample sizes, heterogeneity between cohorts, and limited large scale validation mean that performance remains variable, with reported accuracies typically in the 0.60–0.77 range.

These eight studies collectively demonstrate that MRI—specifically through DCE-MRI, T2-weighted radiomics, and DWI—provides a non-invasive, preoperative alternative to invasive histological assessment for predicting treatment response in osteosarcoma. Machine learning integration, utilizing random forest, SVM, and logistic regression, has consistently enhanced predictive performance with accuracies between 0.84 and 0.89. Furthermore, multi-parametric MRI approaches combining T2WI, DCE-MRI, and DWI sequences with clinical features have been shown to outperform single-parameter methods, offering clear utility for surgical planning, treatment modification, and survival prediction. However, these studies face persistent limitations, including small sample sizes (ranging from 12 to 209 patients), significant methodological heterogeneity in ROI selection and validation, and a lack of external validation, all of which hinder clinical translation. A key contradiction exists in the literature: while some studies successfully utilize volumetric and radiomic features to predict chemotherapy response, O. Yildirim et al. concluded that traditional tumor volume measurements are unreliable for predicting histological necrosis [46].

### Transformer

**Fig 6:**
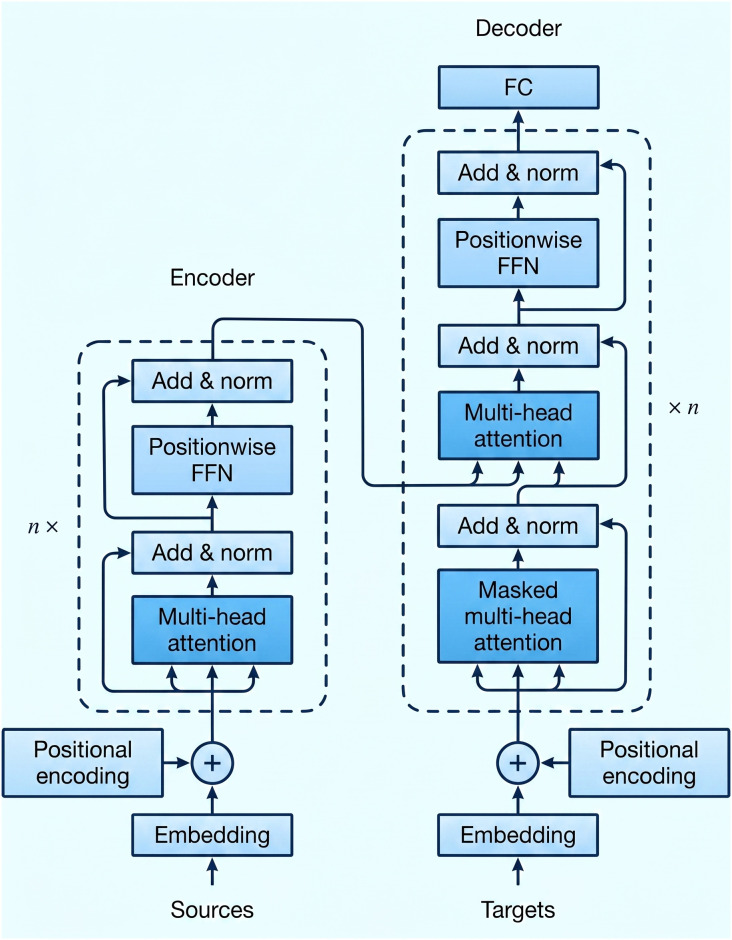
Transformer model.

The Vision Transformer (ViT) is a transformer architecture adapted to image analysis. Instead of scanning the image with convolutions, ViT divides an image into fixed size patches, embeds these patches as a sequence (analogous to word tokens in language models), and then processes them with self attention layers. When trained on sufficiently large image collections, ViT achieves competitive or superior performance in classification and detection tasks compared with conventional CNNs, and has inspired many hybrid designs that combine transformers with convolutional backbones. A schematic overview of the basic transformer structure is shown in Fig 6.

Liu et al. introduced OSTransnet, a segmentation framework that combines a transformer module with a U Net backbone for osteosarcoma MRI. The pipeline begins with standard pre processing, including denoising and intensity normalization, and then applies a transformer block to model broad spatial relationships in the image, while U Net is used to recover precise tumor contours, particularly at indistinct margins. A multi scale fusion strategy is employed to merge features from different resolution levels, and the network is trained on expert annotated datasets with cross validation. Their results show stable performance and more accurate delineation of tumor regions compared with conventional architectures [51]. Ouyang et al. proposed a compact U Net–style encoder–decoder network augmented with a multilevel guided self aware attention module for segmenting osteosarcoma on MRI. The attention component, inspired by transformer self attention, emphasizes informative regions and suppresses background responses, enabling the model to capture image wide context while ignoring irrelevant structures. They further refine the dataset organization to simplify the learning task and lower computational cost. Overall, the model attains better segmentation quality with fewer parameters, making it attractive for use in environments with limited hardware resources [52]. Ling et al. designed a new segmentation scheme that integrates transformer blocks with convolutional neural networks in a sequential (serial) manner for osteosarcoma MRI analysis. In their design, a transformer stage first extracts global representations, after which convolutional layers focus on local refinement and detailed boundary modeling. This serial fusion of global and local processing yields strong performance, with a reported Dice coefficient of 0.924 and Intersection over Union of 0.868. Tests against parallel and mixed fusion variants show that the serial configuration provides the best balance of accuracy and stability [53]. Zou et al. [54] presented RTUNet++, a hybrid CNN–transformer model for osteosarcoma MRI segmentation that combines ResNet residual blocks, transformer based attention, and the densely connected skip pathways of UNet++. The encoder captures rich spatial features, while the dense skip connections in the decoder merge information across multiple resolution levels, allowing simultaneous use of coarse global cues and fine local detail. Comparative experiments against standard segmentation networks show that RTUNet++ achieves higher accuracy, indicating its promise as a tool to support radiological assessment in clinical workflows. He et al. developed a multi scale feature enhancement network (MFENet) tailored to osteosarcoma MRI segmentation [55]. In this approach, transformer components are used to represent broad contextual information, whereas convolutional modules focus on detailed, small scale structures. These representations from different scales are then combined by specialized fusion blocks that suppress noise and sharpen tumor borders. Additional edge focused residual units further improve contour quality and help prevent overfitting when only limited training data are available, enabling reliable automatic segmentation in small sample settings.

These five studies collectively demonstrate a convergent trend toward hybrid CNN-Transformer architectures, such as OSTransnet, UATransNet, MFENet, and RTUNet++, which integrate attention mechanisms into U-Net frameworks to resolve the persistent challenges of fuzzy tumor boundaries and morphological variability in osteosarcoma MRI images. Technical innovations are diverse and highly specific, ranging from edge-enhancement modules (BAB) and combined loss functions in OSTransnet, to UATransNet’s lightweight design using multilevel guided self-aware attention that achieves IOU/DSC values of 0.922/0.921 on datasets exceeding 80,000 images. MFENet provides a few-shot learning capability through multi-scale feature enhancement and prior mask integration, while RTUNet++ leverages ResNet residual modules combined with dense skip connections to enable flexible feature fusion. Furthermore, the introduction of objective quantification methodologies provides clinicians with essential area measurements for enhanced diagnostic reference. Despite these advances, all studies remain constrained by fundamental clinical and technical barriers, including the high noise and blurred edges in MRI images, significant morphological variability, and the inherent difficulty of manual segmentation for clinicians in resource-limited settings. These models also consistently struggle with overfitting caused by data noise and face the ongoing challenge of bridging the gap between high research performance and practical clinical implementation.

### Assessment of Reporting Bias

We did not perform a meta-analysis, and most of the included studies reported only a narrow range of performance measures. For that reason, formal statistical evaluations of reporting or publication bias—such as inspection of funnel plot asymmetry or tests for small study effects—were not undertaken. Instead, to lessen the likelihood of omitting relevant studies, we applied a deliberately broad search strategy in two major bibliographic databases and complemented this with both backward (reference list) and forward (citation) searching.

### Certainty of Evidence

We also chose not to rate the certainty of evidence using structured frameworks such as GRADE. The studies in this review cover diverse AI model families, MRI acquisition protocols, and outcome definitions, which makes it difficult to apply a single quantitative grading system in a consistent and interpretable way. Rather, for each application area we evaluated the evidence base qualitatively, considering aspects such as study design, sample size, the presence or absence of external validation, and how closely the reported outcomes aligned with clinically meaningful endpoints.

### Ethical Considerations

Ethical terrain must be negotiated when dealing with the use of AI in osteosarcoma care. One of the main concerns is algorithmic bias, as models that are primarily built on a specific geographic or demographic group might have different performance on smaller populations of the group that could potentially increase existing healthcare disparities. Moreover, the “black box” aspect of deep learning makes clinical accountability and informed patient consent for the algorithm more difficult to explain to clinicians, since it is hard to explain exactly what features an algorithm would look at to make a diagnosis or recommendation for surgery. Before these tools can be ethically incorporated into clinical practice, it is imperative to have clear and explainable AI (XAI) frameworks and to implement strict patient data privacy protections.

## Results and Discussion

### Datasets Descriptions

This study describes 38 different studies which used different medical imaging datasets focused on osteosarcoma research. The datasets primarily include MRI (T1, T2, DWI, DCE-MRI), along with some multi-modality and clinical data. They vary in size, preprocessing methods, and annotations, supporting tasks like tumor segmentation, treatment response analysis, and AI model development. Each dataset is briefly summarized with its key features for easy reference. Table 2 presents all the dataset descriptions of previous studies.

In the Table 2, the study [33] and [41] is not included because, [33] only used one single image chosen arbitrarily and [41] does not use specify particular dataset rather it mixes 5 different dataset from different sources without standardized method and just did random experiment on different train-test split while the dataset is not mad public.

### Risk of Bias Analysis

In medical domain, depending on the patient data some risk factor might be present in the research. These biases often hamper the generalizability of the research and put a question mark on the credibility of the research. Table 3 shows the risk of bias factors.

Table 3 summarizes the risk-of-bias assessment for the 38 included studies across key methodological domains, including patient selection, imaging acquisition and annotation, model development and validation, and reporting transparency. Overall, most studies were judged to have some concerns or high risk of bias in at least one domain, largely due to small, single-centre samples, retrospective study designs, and limited or absent external validation. In many articles, details on blinding of image annotators, handling of missing data, and pre-specification of analysis pipelines were poorly reported, making the true risk of bias difficult to evaluate. By contrast, descriptions of model architectures and performance metrics were generally more complete. Only a minority of studies achieved a low risk of bias across all domains, highlighting the need for future work to adopt prospective, multi-centre designs, standardized MRI protocols, and transparent reporting frameworks to strengthen the evidence base for AI-assisted MRI applications in osteosarcoma. The criteria of being ‘LOW’, ‘MODERATE’ and ‘HIGH’ is given below:

Participants Risk of Bias This evaluates the patient cohort’s representation and size. A ‘LOW’ risk utilizes large, multi-center cohorts (>200 patients) with strict selection criteria. A ‘MODERATE’ risk involves moderately sized retrospective cohorts (50–200 patients) from one or a few centers, introducing potential selection bias. A ‘HIGH’ risk relies on extremely small samples (<50 patients) or convenience sampling, severely limiting generalizability.

Predictors / Imaging Risk of Bias This assesses MRI data acquisition, standardization, and preprocessing. A ‘LOW’ risk applies strictly standardized protocols and robust harmonization to eliminate scanner variations. A ‘MODERATE’ risk involves standard clinical scans with inherent scanner variability mitigated by basic preprocessing. A ‘HIGH’ risk features high heterogeneity in imaging protocols without adequate preprocessing, leaving models susceptible to scanner noise.

Outcome / Reference Risk of Bias This evaluates the reliability of the ground truth. A ‘LOW’ risk uses hard, objective clinical endpoints, like survival rates or histopathologically confirmed tumor necrosis ≥90%. A ‘MODERATE’ risk relies on manual radiologist segmentations, which carry inherent subjective variability. A ‘HIGH’ risk uses poorly defined references lacking expert consensus or relies solely on unverified automated software.

Analysis Risk of Bias This scrutinizes the model validation strategy and risk of data leakage. A ‘LOW’ risk employs robust internal validation alongside a strictly independent external cohort. A ‘MODERATE’ risk uses sound internal validation and strict patient-level data splitting but lacks external validation. A ‘HIGH’ risk exhibits severe data leakage, such as splitting data at the 2D slice level instead of the patient level, artificially inflating accuracy.

Overall Risk of Bias This represents the cumulative assessment of methodological quality. A ‘LOW’ overall risk requires a ‘LOW’ rating across all key domains. A ‘MODERATE’ overall risk applies if one or more domains score ‘MODERATE’, provided none are rated as ‘HIGH’. A ‘HIGH’ overall risk means the study scored ‘HIGH’ in at least one domain, significantly compromising the reliability of its findings.

### Performance Metrices Analysis

Magnetic resonance imaging (MRI) is central to diagnosing osteosarcoma and planning treatment, as it offers detailed visualization of tumor shape, local extent, and treatment response. With the growing use of deep learning and radiomics-based methods in image analysis, it has become essential to judge their performance using robust, clinically meaningful evaluation measures. Commonly applied indices include accuracy, sensitivity, specificity, precision, recall, F1 score, and the area under the receiver operating characteristic curve (AUC ROC), which are used to evaluate models for segmentation, classification, and outcome prediction. For segmentation in particular, spatial overlap metrics such as the Dice similarity coefficient (DSC) and Intersection over Union (IoU) are widely reported to quantify how closely automated tumor contours match expert reference annotations. Careful interpretation of these metrics not only allows direct comparison between different approaches but also helps to reveal practical issues such as under segmentation (missed tumor portions) or over segmentation (inclusion of non tumor tissue). A thorough understanding of these indicators enables researchers and clinicians to appreciate how models behave across a range of scenarios, from early tumor detection to prediction of histopathological response to neoadjuvant chemotherapy. The present section reviews comparative performance across osteosarcoma MRI datasets and methods, emphasizing their advantages, limitations, and implications for routine clinical use. The definitions of all performance measures reported in previous studies are summarized in Table 4.

**Table 4:** Performance metrices.

Approach	Number of Studies	Included Studies	Dice(%)	IoU(%)	Accuracy (%)	AUC(%)
Attention	5	[18–22]	64.6–98.5	47.8–97.1	83.9–99.5	N/R
Clustering	5	[23–27]	81.6–89.8	70.8–83.0	92.0–98.0	N/R
CNN	6	[28–33]	91.4–96.4	86.2–92.8	91.0–95.5	95.0
Lightweight	5	[34–38]	91.4–99.4	85.3–88.5	93.7–99.4	N/R
Multimodal	4	[39–42]	94.5–94.9	89.8–91.5	91.2–99.7	94.3
Radiomics	8	[43–50]	N/R	N/R	76.5–95.0	73.9–97.0
Transformer	5	[51–55]	90.5–94.9	86.8–91.8	99.6	N/R

**Notes:** N/R = not reported; IoU = Intersection over Union; AUC = Area Under the Curve.

There are clear differences in the stability and effectiveness of the segmentation with respect to different methodological approaches when comparing their segmentation performance. The multi-modal architecture and CNN architectures most effectively and also consistently performed. The CNNs achieved a narrow range between 91.4 and 96.4 intermediate Dice coefficient and 86.2–92.8 intermediate Intersection over Union (IoU) accuracy results. The multimodal approaches essentially had the lowest Dice variance (94.5–94.9%) and high accuracy (91.2–99.7%), indicating that combinations of different data types yield very stable segmentation results. Results revealed excellent performance, with Dice scores (90.5–94.9%) comparable to CNNs, and the highest accuracy of 99.6%, for Transformers. On the opposite side, traditional Clustering approaches were far behind deep learning architectures in terms of upper-bound Dice score (81.6–89.8%) and IoU (70.8–83.0%), showing low performance. Like the voering, Radiomics approaches generally failed to report overlap metrics such as Dice and IoU, suggesting a lack in ability to segment pixel-wise on the boundaries as opposed to using only radiomic features. Variance in Attention and Lightweight Models An interesting result is the fact that the models with Attention mechanisms separated from the rest of the model showed the highest deviation in the literature. They can outperform even Dunman with nearly the perfect Dice scores (98.5%) but have the lowest minimums in the data set (Dice: 64.6%, IoU: 47.8%). The joints are clearly quite sensitive to certain data properties, or have to be heavily tuned with hyperparameters for stability, as evidenced by this huge spread. The lowest absolute peak Dice score reviewed was 99.4% for lightweight models that generally maintained a high accuracy (93.7–99.4%) indicating that representative computational efficiency did not require compromises in segmentation ability.

### Clinical Relevance of Performance Metrics in Osteosarcoma Diagnosis

Standard quantitative indices such as Intersection over Union (IoU), Dice coefficient, accuracy, F1 score, area under the receiver operating characteristic curve (AUC), sensitivity, specificity, precision, and recall are widely reported in imaging studies, but their true value only emerges when they are interpreted in relation to patient care. High Dice and IoU values in segmentation tasks indicate close agreement between predicted and reference tumor contours on MRI, which is directly relevant for pre operative planning, especially when limb preserving surgery is considered and negative margins are critical. Sensitivity (recall) reflects how reliably the method detects true tumor regions or positive cases; in clinical terms, insufficient sensitivity risks missed lesions, delayed diagnosis, or underestimation of tumor extent. Specificity, conversely, describes how well normal tissue is recognized as non tumor, helping to avoid unnecessary biopsies, overtreatment, or excessive resection of healthy structures. Precision (positive predictive value) is particularly important when a positive finding leads to invasive or toxic interventions, as low precision corresponds to a higher proportion of false positives and potentially avoidable procedures.

Overall accuracy offers a global summary of performance but can be misleading in highly imbalanced datasets, which are typical for medical images where non tumor voxels far outnumber tumor voxels. In such settings, combined indices like the F1 score, which harmonizes precision and recall, provide a more informative picture of clinically meaningful performance. The AUC of the ROC curve characterizes the ability of a model to distinguish between classes across a range of thresholds; a high AUC suggests robust discrimination, which is essential for risk stratification and for integrating model outputs into decision support tools. Ultimately, these numerical measures need to be interpreted against concrete clinical objectives—earlier and more reliable diagnosis, fewer diagnostic errors, improved prognosis, and more appropriate treatment selection. For this reason, future work should not only aim to optimize metric values on retrospective datasets, but also to test these methods prospectively in real clinical workflows and to link performance to patient level outcomes to ensure true clinical usefulness.

**Fig 7:**
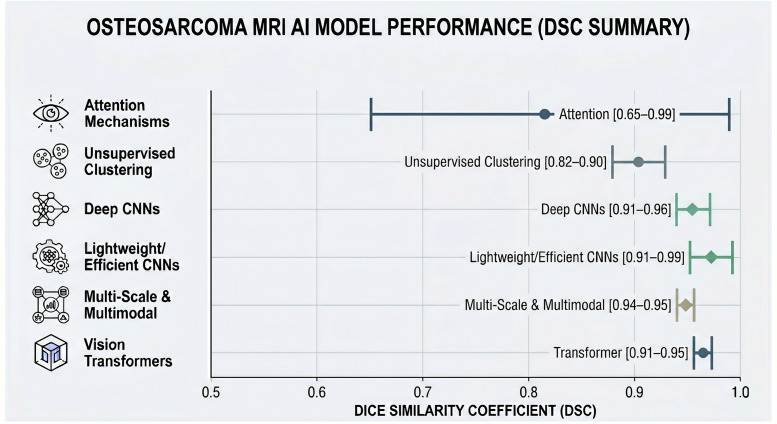
Statistics of different aspects of research.

To systematically address the comparative performance of the AI models and provide a quantitative summary (Fig 7), we stratified the Dice scores by model type. An analysis of the reported Dice performance ranges reveals distinct accuracy profiles across the categories, with the mean values (calculated from the upper and lower bounds of the reported ranges) highlighting the strengths of specific architectures. Lightweight networks demonstrated the mean Dice score of 91.9% (range: 91.4–99.4%). Multimodal approaches demonstrated the highest value for DSC with a mean of 94.7% (range: 94.5–94.9%) and standard CNNs with a mean of 93.3% (range: 91.4–96.4%). Transformer models also showed robust spatial overlap with a mean Dice of 92.1% (range: 90.5–94.9%). In contrast, Unsupervised Clustering yielded a mean of 85.7% (range: 81.6–89.8%), while Attention Mechanisms exhibited the widest performance variance, resulting in a mean of 87.67% (range: 64.6–98.5%).

Notably, the Radiomics category was excluded from this quantitative Dice summary and the associated graph. This exclusion is because radiomics models are designed to evaluate prognostic clinical endpoints (such as survival outcomes or chemotherapy efficacy) rather than spatial tumor delineation; consequently, Dice similarity coefficients are entirely not reported (N/R) within these specific studies.

### Insights From Previous Studies

Table 5 presents the summary insights of previous studies.

**Table 5:** Summarized descriptions of previous studies.

Model Type / Approach	Representative Studies	Typical Dataset Size (Patients / Images)	Validation Method	Best Metric (approx.)
Attention Mechanisms (e.g., Self-Attention, Axial Attention)	[18–22]	40–204 patients; 3,255 to >80,000 images	5-fold cross-validation; 70/30 or 80/20 train/test splits	DSC: 0.95–0.96; AUC: 0.93
Unsupervised Clustering (e.g., FCM, K-Means, Otsu)	[23–27]	7–55 patients; 50–3,520 images/slices	Internal train/test splits; direct comparison to manual ground truth ROIs	Accuracy: 0.92–0.98; DSC: 0.70–0.83
Deep CNNs (e.g., DecoupleSegNet, DFANet)	[28–33]	136–204 patients; > 4,000 to >80,000 images	5-fold / 10-fold cross-validation; 70/30 or 80/20 patient-level splits	DSC: 0.92–0.96; IoU: 0.88–0.90
Lightweight / Efficient CNNs (e.g., SepUNet, RFN, ACRNet)	[34–38]	198–286 patients; > 70,000 to >80,000 images	10-fold cross-validation; 70/30 or 80/20 train/test splits	DSC: 0.89–0.94; Params: 2.33M–20.3M
Multi-Scale & Multimodal (e.g., PESNet, TBNet)	[39–42]	78–204 patients; 10–40 slices/sequence to >80,000 images	5-fold / 10-fold cross-validation; internal train/test splits	DSC: 0.94–0.95; AUC: 0.82–0.95
Machine Learning Radiomics (e.g., LASSO, SVM, Cox models)	[43–50]	12–209 patients; tissue-level ROIs, DCE-MRI, or mpMRI	5-fold cross-validation; independent external validation cohorts	AUC: 0.80–0.95; C-index: 0.74–0.76
Vision Transformers (e.g., UATransNet, RTUNet++)	[51–55]	40–286 patients; 274 to >80,000 images	K-fold cross-validation; 70/30 or 80/20 train/test splits	DSC: 0.82–0.94; IoU: 0.86–0.90

## Current Trends and Applications

**Fig 8:**
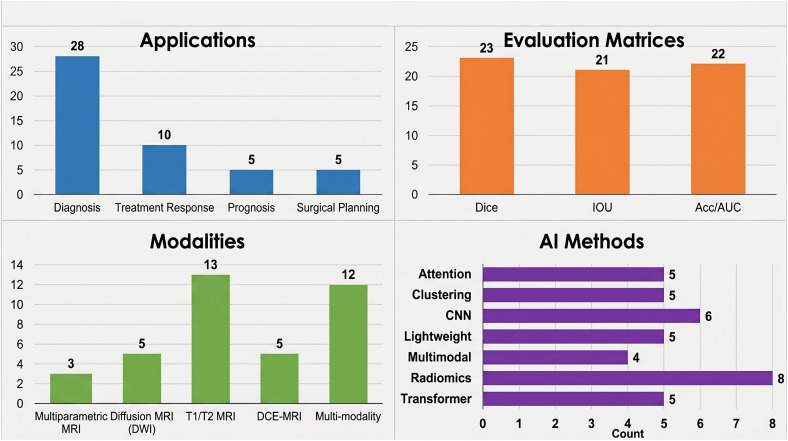
Statistics of different aspects of research.

As illustrated in Fig 8, current work on MRI-based computational methods for osteosarcoma is distributed very unevenly across research themes, with several important domains still relatively neglected. Most published studies concentrate on diagnostic tasks, whereas questions related to prognosis, individualized treatment planning, and intraoperative or surgical guidance receive far less attention. Likewise, evaluation is typically framed in terms of image-based segmentation scores such as the Dice coefficient, with only limited use of clinically grounded endpoints such as RECIST measurements or direct comparison with histopathology. This disconnect highlights a persistent gap between technical performance and true clinical impact.

On the imaging side, multiparametric and diffusion-weighted MRI are frequently employed, but genuine multimodality integration (for example, combining MRI with CT, PET, or radiographs) remains uncommon, restricting the potential benefits of cross-modal information. Methodologically, most work is still built around classical radiomics pipelines and convolutional neural networks, while more recent architectures such as transformer-based models and sophisticated multimodal fusion strategies are used only sporadically, likely reflecting restrictions in dataset size and computational resources.

From a study-design perspective, the field is dominated by retrospective, single-centre analyses with limited testing in real-world settings, which raises concerns about generalizability. Clinicians are often involved mainly in tasks such as annotation and region-of-interest definition, but only a small proportion of models are prospectively evaluated within routine clinical workflows with active clinician feedback. The scarcity of prospective trials and multi-centre collaborations further increases the risk that models become tuned to local data idiosyncrasies and fail when transferred to new institutions. Finally, issues of runtime and hardware efficiency are rarely treated as priorities, even though these factors are critical for deployment in time-sensitive or resource-constrained environments. Overall, progress in MRI-based osteosarcoma research now needs to shift from narrowly optimizing numerical performance metrics toward developing clinically meaningful, computationally efficient, and scalable tools that can be seamlessly embedded in everyday healthcare practice.

### Limitations and Future Directions

While the integration of deep learning (DL) approaches into osteosarcoma imaging has demonstrated considerable promise, several persistent limitations hinder their widespread clinical translation. A critical evaluation of current research highlights five key challenges that must be addressed in future investigations:

### Risk of Bias in Included Studies

Overall, the methodological quality of the included studies was variable and frequently limited by features that increase the risk of bias. Most investigations were retrospective, single centre analyses with relatively small sample sizes and limited reporting of patient selection procedures or missing data handling. External validation on independent institutional datasets was uncommon, and hyper parameter tuning strategies were often incompletely described. Ground truth tumour annotations were typically generated by one or two radiologists, but inter observer variability and blinding to clinical data were rarely reported. Collectively, these issues suggest a moderate to high risk of bias in many studies, particularly in relation to patient selection, overfitting, and applicability to broader clinical populations.

### Data Scarcity

One of the most pressing limitations in osteosarcoma-focused DL research is the limited availability of large, high-quality, annotated imaging datasets. Osteosarcoma is a relatively rare malignancy, and data acquisition is further complicated by institutional variability in imaging protocols and ethical constraints surrounding patient data sharing. As a result, many DL models are developed and validated on small or institution-specific datasets, leading to overfitting and reduced generalizability. To overcome this barrier, future studies should prioritize the development of multi-center, open-access imaging repositories that include detailed clinical annotations, histopathological outcomes, and longitudinal follow-ups. Federated learning and data harmonization techniques also hold potential in facilitating model training across decentralized data sources without compromising patient privacy.

### Limited Evaluation Metrices (Algorithmic vs. Clinical Validation)

Most current studies report performance metrics such as Dice Similarity Coefficient, Intersection over Union (IoU), accuracy, and AUC, which are valuable from a computational perspective. However, these algorithmic metrics do not fully capture the clinical relevance or utility of a model. There remains a lack of standardized clinical validation protocols to assess how AI-generated outputs influence diagnostic accuracy, therapeutic decisions, or patient outcomes. Future research should incorporate clinically interpretable endpoints and engage in prospective validation studies. Integration with radiological workflows and comparison with expert-level interpretations will be crucial to establishing clinical credibility.

Many studies reported only the best performing configuration without presenting negative or intermediate results, and confidence intervals or repeated run variability were seldom provided. Because we did not conduct formal statistical tests for reporting bias and could not access unpublished models with suboptimal performance, the presence and extent of reporting bias in this literature remain uncertain and should be considered when interpreting the synthesized findings.

### Problem-specific Algorithm

Not all algorithms or AI models work well for every single type of image. For example, one particular algorithm may generate good results for MRI images related to a particular disease where it doesn’t perform well for MRI images of other diseases. This is mainly because, even though they are all MRI images, different diseases have different patterns in intracellular environments, and also, different staining chemicals are used for identifying different aspects or elements of the body. This is why, in the future, there is much scope to develop an osteosarcoma-specific algorithm that works for osteosarcoma-related MRI images.

### Heterogeneity Causes Unstandardized Evaluation

Because of the large heterogeneity among the 38 included studies in terms of methods and clinical context, we did not choose to perform a formal quantitative statistical pooling (e.g., calculation of an I2 statistic). The assessment methods vary significantly among the algorithmic methods: Unsupervised Clustering, Deep CNNs and Vision Transformers, making a major emphasis on the static spatial overlap metrics: Dice Similarity Coefficient (DSC), pixel-wise accuracy and Intersection over Union (IOU). Lightweight/Efficient CNNs include computational endpoints, trading-off between DSC and count of parameters and Floating Point Operation (FLOFs). Besides, Attention Mechanisms and Multi-Scale/Multimodal Networks are often trained to combine spatial DSC with the Area Under the curve (AUC) for tumor classification, while Machine Learning Radiomics are based on the idea to remove spatial segmentation and design longitudinal clinical outcome metrics based on AUC, Kaplan-Meier analyses, and the Concordance index (C-index). The primary endpoints in the different categories have huge ranges of scores from mere boundary overlap for a static tumor to the hardware efficiency or the prognosis for survival, so an aggregate heterogeneity statistic across these wide-scoped differences would be mathematically invalid and draw inconclusive conclusions.

### Black Box Nature of AI

Despite their high predictive performance, many deep learning models, especially convolutional neural networks and transformer-based architectures, remain inherently opaque. Their “black box” decision-making process limits interpretability, which is a critical requirement for clinical acceptance. Physicians and radiologists need to understand how and why a model makes a specific decision before it can be trusted in patient care. To address this, future models should incorporate explainability tools such as saliency maps, Grad-CAM, or attention heatmaps that highlight regions influencing the model’s output. Additionally, efforts to develop inherently interpretable architectures or rule-based hybrids should be encouraged.

### Limitations in Clinical Translation and Multidisciplinary Involvement

A significant gap in the clinical translation of AI for osteosarcoma stems from the lack of sustained clinician involvement during model design and evaluation. Currently, many image-analysis tools are developed primarily by computer scientists and engineers; as a result, these models often excel on retrospective technical benchmarks but struggle to translate smoothly into everyday clinical decision-making. When radiologists, oncologists, and orthopedic surgeons are not closely involved, critical translational hurdles—such as clinical workflow integration, practical reporting needs, and patient safety considerations—are frequently overlooked, limiting hospital uptake. To successfully bridge the gap from bench to bedside, method development must be genuinely multidisciplinary from the outset. Clinicians are vital to defining clinically meaningful outcomes, curating diverse datasets, shaping how model outputs are presented, and leading the rigorous, prospective clinical trials necessary to validate these tools in real-world healthcare environments.

### Real-world Deployment Constrains

Beyond clinical validation, integrating AI into routine real-world radiological workflows presents substantial logistical challenges. Many advanced architectures, particularly Vision Transformers and highly parameterized Deep CNNs, demand significant computational infrastructure (e.g., high-end GPUs) that is often unavailable in standard hospital settings. Furthermore, for widespread adoption, AI tools must interface seamlessly with existing Picture Archiving and Communication Systems (PACS) and Radiology Information Systems (RIS). Algorithms that require clinicians to export data to separate, standalone software or that otherwise disrupt established diagnostic workflows will inevitably face high friction against clinical adoption.

### Methodological Limitations of This Review

Beyond the limitations of the primary studies, this review has several methodological constraints. First, the search was restricted to publications indexed in Web of Science and Scopus, which may have led to omission of relevant studies in other languages or databases. Grey literature, conference abstracts without full papers, and preprints were not systematically searched. Second, we did not perform a formal tool-based risk-of-bias assessment or certainty-of-evidence grading, and our synthesis was narrative rather than quantitative due to substantial heterogeneity in study designs and outcomes. These factors may introduce residual selection and reporting biases and should be borne in mind when interpreting our conclusions.

Taken together, the overall certainty of evidence supporting AI assisted MRI analysis in osteosarcoma is best regarded as low to moderate. Several segmentation and radiomics models demonstrate high performance on single centre datasets; however, concerns about small sample sizes, retrospective designs, heterogeneous imaging protocols, and scarce external validation substantially limit confidence in their generalisability. Evidence is relatively stronger for segmentation in controlled research settings and more preliminary for prognostic modelling, treatment response prediction, and decision support systems, where only a small number of exploratory studies are available.

## Conclusion

In recent years, machine learning and deep learning have rapidly advanced osteosarcoma imaging analysis, yet a comparative evaluation reveals distinct performance hierarchies among computational strategies. Quantitative synthesis demonstrates that Lightweight and Multimodal Convolutional Neural Networks (CNNs) currently provide the most robust and consistent spatial segmentation, achieving mean Dice Similarity Coefficients exceeding 94%. While standard CNNs and Vision Transformers also exhibit high baseline accuracy, their broader performance variance underscores a heightened sensitivity to dataset scale and annotation quality. Conversely, classical Unsupervised Clustering and generic Attention Mechanisms demonstrate lower or highly variable spatial accuracy, indicating they are less suited for complex tumor delineation. Beyond segmentation, Machine Learning Radiomics models have established a separate, highly valuable clinical niche, consistently proving effective for non-invasive prediction of chemotherapy efficacy and prognostic survival rather than structural boundary detection. Despite these distinct architectural strengths, critical translational gaps remain. A substantial majority of the evaluated models are constrained by small, single-center cohorts, non-standardized imaging protocols, and heterogeneous evaluation metrics, which severely limits their generalizability. Furthermore, the lack of rigorous external validation continues to slow the transition of these algorithms from theoretical research tools to practical clinical applications. Moving forward, progress will depend heavily on the establishment of large, multi-institutional, publicly accessible imaging datasets and universally agreed-upon benchmarking standards. As algorithm selection becomes increasingly tailored to specific clinical endpoints—balancing computational efficiency with predictive power—these AI-driven methodologies possess the clear potential to drive earlier diagnosis, individualize treatment planning, and ultimately improve clinical outcomes for patients with musculoskeletal tumors.

## Supporting information

S1 File Minimal dataset. (XLSX)
